# Collagen XXVII Organises the Pericellular Matrix in the Growth Plate

**DOI:** 10.1371/journal.pone.0029422

**Published:** 2011-12-19

**Authors:** Darren A. Plumb, Laila Ferrara, Tanja Torbica, Lynnette Knowles, Aleksandr Mironov, Karl E. Kadler, Michael D. Briggs, Raymond P. Boot-Handford

**Affiliations:** 1 Wellcome Trust Centre for Cell-Matrix Research, The University of Manchester, Manchester, United Kingdom; 2 Faculty of Life Sciences, The University of Manchester, Manchester, United Kingdom; University of Minho, Portugal

## Abstract

In order to characterise the function of the novel fibrillar type XXVII collagen, a series of mice expressing mutant forms of the collagen were investigated. Mice harboring a glycine to cysteine substitution in the collagenous domain were phenotypically normal when heterozygote and displayed a mild disruption of growth plate architecture in the homozygous state. Mice expressing an 87 amino acid deletion in the collagenous domain of collagen XXVII were phenotypically normal as heterozygotes whereas homozygotes exhibited a severe chondrodysplasia and died perinatally from a lung defect. Animals expressing the 87 amino acid deletion targeted specifically to cartilage were viable but severely dwarfed. The pericellular matrix of proliferative chondrocytes was disrupted and the proliferative cells exhibited a decreased tendency to flatten and form vertical columns. Collagen XXVII plays an important structural role in the pericellular extracellular matrix of the growth plate and is required for the organisation of the proliferative zone.

## Introduction

The classical fibrillar collagens (types I, II, III, V and XI) comprise the major structural elements of the interstitial (ECM) matrix of vertebrates. These collagens share highly conserved C-terminal non-collagenous domains and uninterrupted major collagenous domains of 1011-1017 amino acid residues. At the N-terminus of the major triple helical domain is a short, non-collagenous telopeptide sequence followed by a second much shorter collagenous sequence termed the ‘minor’ helical domain. Finally at the N-terminus of each proα chain there is usually a von Willebrand factor C domain (type A clade genes) or a variable domain flanked by a thrombospondin motif (type B clade genes) (reviewed in [1]). These triple helical molecules co-polymerise to form the cross-striated fibrils apparent in connective tissues when negatively stained and viewed by electron microscopy [2], [3]. Type XXVII collagen is a novel member of the fibrillar collagen gene family [4], [5]. This homotrimeric collagen, together with the closely related type XXIV collagen [6], differ from the classical fibrillar collagens in several notable respects. The major triple helical domains of these novel members of the fibrillar collagen family are shorter than their classical counterparts being 991–997 amino acid residues in length. Type XXIV and XXVII collagens have two interruptions in the characteristic collagen Gly-X-Y repeat at conserved locations in their major helical domains. In addition, types XXIV and XXVII collagen lack the N-terminal telopeptide region and the N-terminal minor helical domain that characterise the classical fibrillar collagens. The N-terminus of both novel types of fibrillar collagen consists of a ‘variable’ domain and a thrombospondin domain similar to that of the type B clade genes. Phylogenetic analysis reveals that types XXVII and XXIV collagen form a distinct clade (named type C) within the fibrillar collagen family [4]–[6].

Type XXVII collagen is expressed in a variety of tissues during development including skin, stomach, gonad, lung, aorta and tooth but its most prominent expression is in cartilage [4], [5], [7]. Expression is particularly high in proliferative zone chondrocytes of the epiphyseal growth plate [7], [8]. The SOX9 [9] and Lc-Maf [10] transcription factors have been shown to control chondrocyte expression of type XXVII collagen. Immunolocalisation of type XXVII collagen in the skeleton revealed weak pericellular staining around articular chondrocytes and in the growth plate, stronger staining in the matrix surrounding proliferative chondrocytes that became intense as the matrix around hypertrophic chondrocytes condensed [7], [8]. Immuno-electron microscopy of cartilage extracts revealed that type XXVII collagen appears to form thin non-striated fibrils perhaps organised in a network but certainly distinct from the cross-striated fibrils formed by the classical fibrillar collagens [7], [8].

In order to characterise further the function of type XXVII collagen, we generated a series of mice expressing mutant forms of type XXVII collagen. We decided to introduce mutations into the collagen XXVII gene rather than knock it out for the following reasons: Firstly, we believed that another group was already making a knockout of collagen XXVII although it subsequently transpired that they were working on a different gene. Secondly, straight knockouts of other fibrillar collagen genes (e.g. *Col1a1*) had not necessarily provided a particularly informative insight into the genes function due to early embryonic lethality. Thirdly, a longer-term aim of these studies is to identify and investigate human disease(s) caused by mutations in collagen XXVII. For other fibrillar collagens, these types of disease are far more commonly caused by missense rather than null mutations. Finally, the mutation strategy adopted to design the targeting construct allowed the production of two mutant forms of collagen XXVII from a single construct, namely a Gly to Cys mutation in the collagenous domain and separately, an 87 amino acid deletion in the collagenous domain. The latter deletion we believed was sufficiently severe to produce a functional null for collagen XXVII although our subsequent data indicated that this was not the case. We demonstrate the introduction of a Gly to Cys substitution (G1516C) within the triple helical domain, a type of mutation that has significant pathogenic effects when present in the classical fibrillar collagens (see [11]), had little phenotypic effect with mutant type XXVII collagen secretion appearing unaffected. In contrast, mice homozygous for an 87 amino acid deletion in the collagenous domain had skeletal abnormalities, a chondrodysplasia and died at birth because of a lung defect. Mice homozygous for the 87 amino acid deletion targeted specifically to cartilage survived into adulthood but were severely dwarfed. Immunohistochemical and EM analyses suggested that type XXVII collagen plays an important role in regulating the organization of the pericellular matrix in the growth plate.

## Results

### Gene targeting

**Figure 1:**
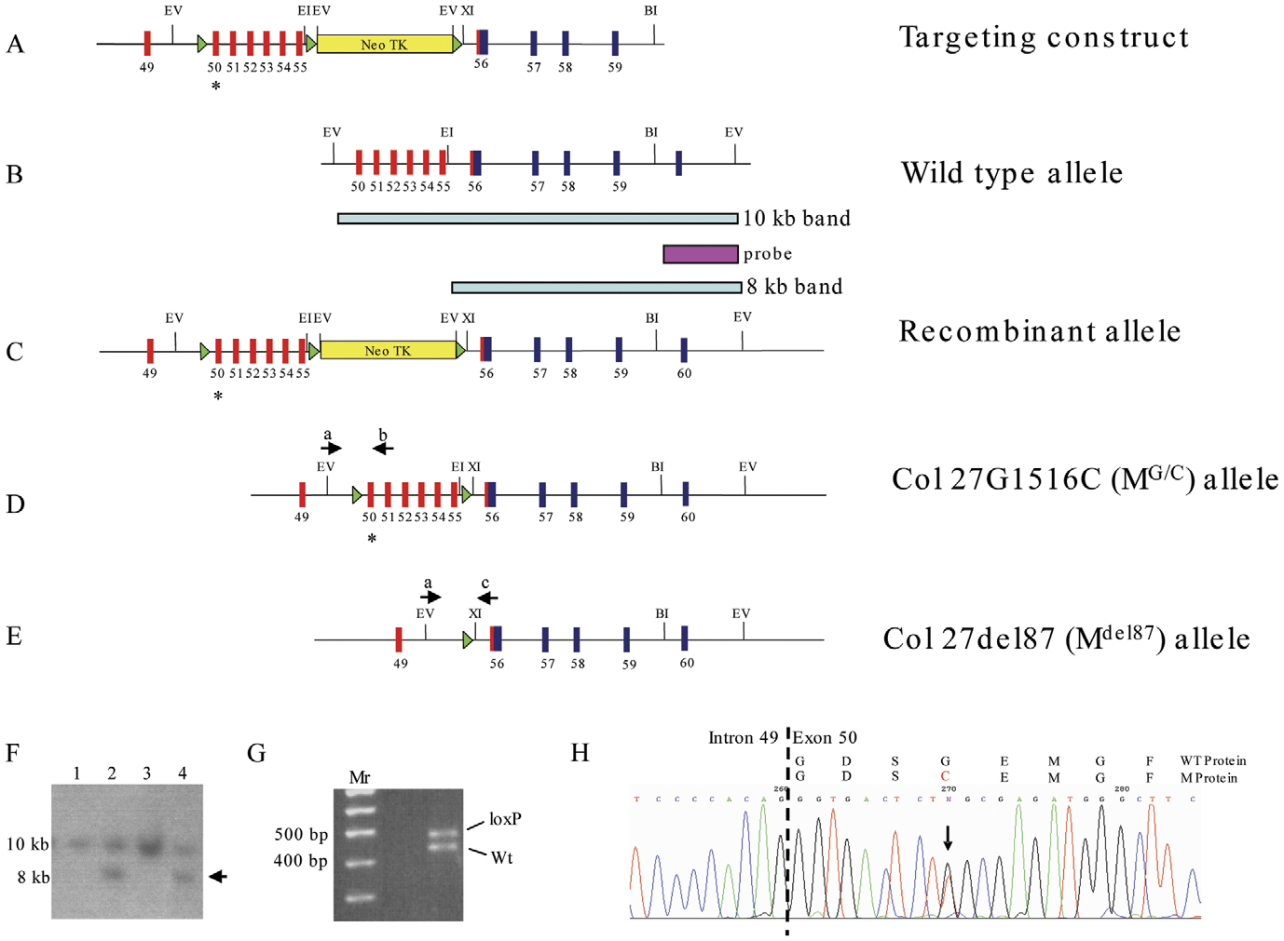
Schematic of the gene targeting strategy used to generate collagen XXVII mutant mice. A. The targeting construct with red numbered boxes representing exons at the C-terminus of the Gly-X-Y domain and blue boxes representing exons in the NC1 domain of collagen XXVII. Green triangles represent loxP sites and the yellow rectangle a neotk selection cassette. Asterisk represents the site of introduced mutation in exon 50. B. Wild-type allele. C. Modified collagen XXVII locus after homologous recombination. The external probe detects a 10 kb Eco RV band from the wild type allele and an 8 kb band from the mutant allele on Southern analysis (see F). D. The Col27G1516C allele after cre*-*mediated removal of the neotk­ cassette. E. The Col27G1516C mouse line was crossed with the cre-deletor mouse to generate the Col27del87 allele. Alternatively, the Col27G1516C line was crossed with a col2-cre line to produce the Col27delskel mouse in which the 87 amino acid deletion is only present in cells that have expressed collagen II. The position of primers a and c (see methods for sequences) used for genotyping this allele are shown F. Southern blot of Eco RV (EV) cut DNA extracted from homologously recombined clones (lanes 2 and 4) containing the 8 kb targeted allele (arrow). G. PCR analysis of DNA from recombined clone using primers ‘a’ and ‘b’ (Fig. 1 D) showing 5′ most loxP site incorporation by presence of 500 bp band (loxP) in addition to wild type 460 bp band (Wt). H. Direct sequencing of PCR product amplified from genomic DNA of targeted ES cells demonstrating the presence of both the wild type and Col27G1516C alleles.

Approximately 330 G418-resistant ES clones that had been transfected with the collagen XXVII targeting vector (Fig. 1 A) were analysed for homologous recombination by Southern blotting of EcoRV-cut genomic DNA. Nine clones were found to have homologously recombined through the appearance of an 8 kb band in addition to the wild-type band at 10 kb (Fig. 1 C & F). Furthermore, all of these clones were also shown to contain the loxP site in intron 49 (Fig. 1 G) and the mutation in exon 50 encoding the G1516C substitution (Fig. 1 H). Two correctly targeted ES cell clones were grown up and transiently transfected with a cre recombinase expression vector to delete the floxed selection cassette and produce the Col27G1516C (M^G/C^) allele (Fig. 1 D). Two independently-targeted ES cell clones were used to generate germline-transmitting chimeras from which all subsequent mutant collagen XXVII lines were established.

### A Gly to Cys substitution in the type XXVII collagenous domain has little phenotypic effect

**Figure 2:**
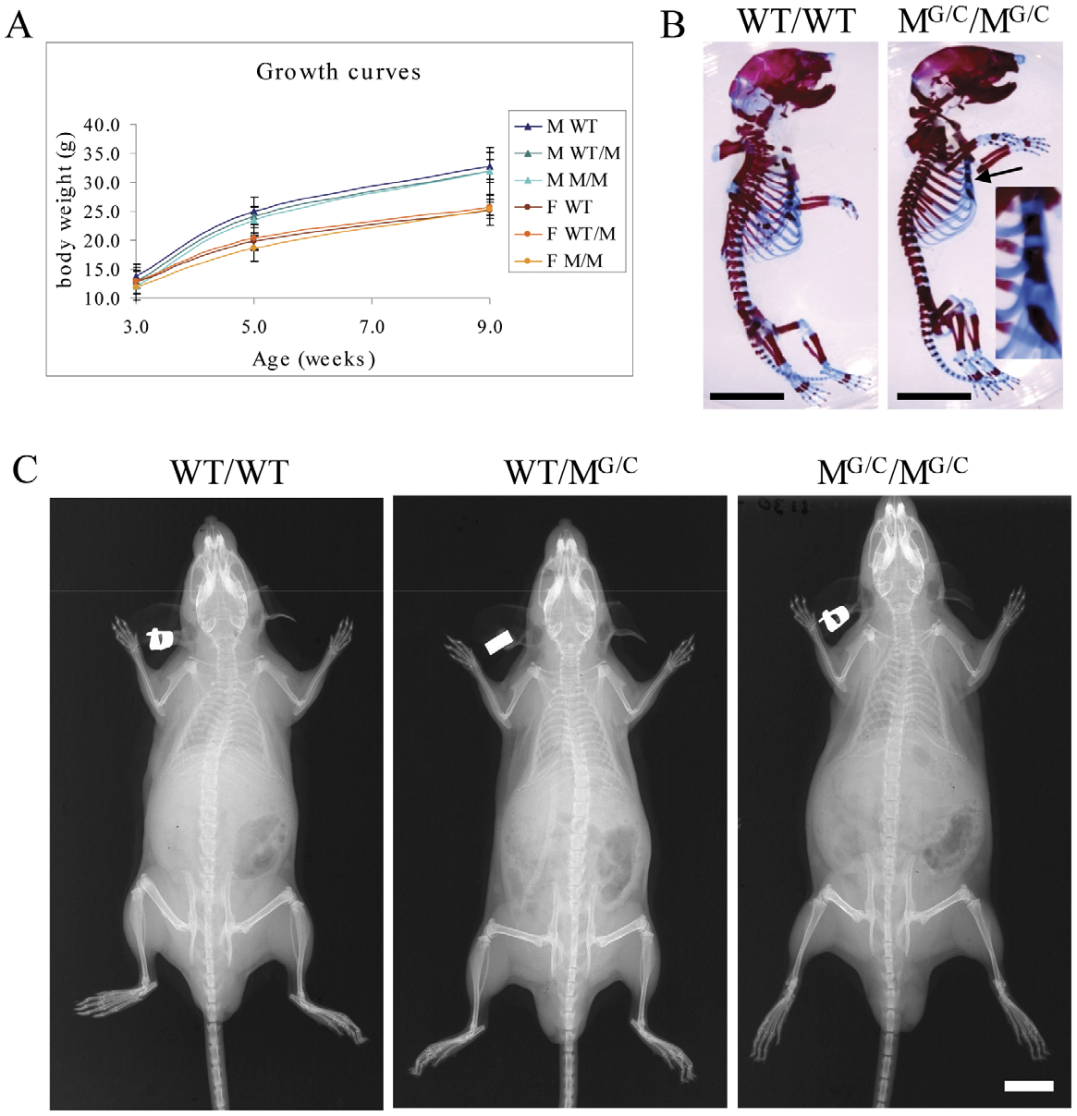
Col27G1516C mice have no gross phenotype. A. Growth curves for the offspring of heterozygous crosses. B. Skeletal preps of new born mice revealed a fusion of the 4^th^ and 5^th^ sternebrae (arrow and see inset) in mice homozygous for the mutation when compared with wild type littermates but no other differences. Scale bar  =  5 mm C. X-ray analysis of 9 week old mice revealed no obvious phenotype of the skeleton in mutant mice. Scale bar  =  10 mm. Wild type (WT/WT), Heterozygous (WT/M^G/C^), Homozygous (M^G/C^/M^G/C^).

**Figure 3:**
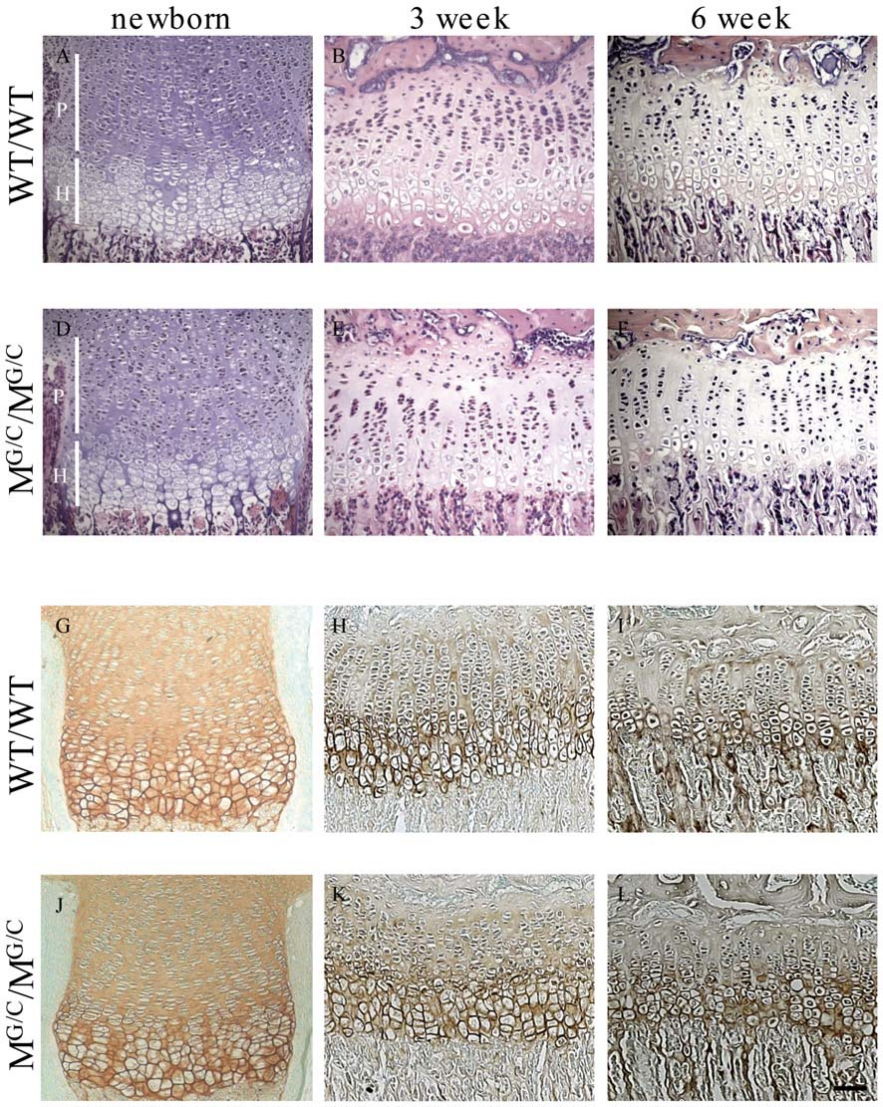
Histological analyses of Col27G1516C mouse growth plate. H & E staining of tibial growth plates of new born, 3 and 6 week (A&D, B&E and C&F respectively) wild type (WT/WT) mice and mice homozygous for the Col27G1516C allele (M^G/C^/M^G/C^). M^G/C^/M^G/C^ mice sometimes exhibit a slightly disordered proliferative (P) zone which is apparent in 3 (E) but not 6 (F) week sample. Immunolocalisation of collagen XXVII in M^G/C^/M^G/C^ mice is normal in the proliferative (P) and hypertrophic (H) zone of tibial growth plates in new born, 3 and 6 week (J, K and L, respectively) when compared with the wild type littermates (WT/WT - G, H, I). Scale bar in (L)  =  100 µm.

Mice heterozygote (WT/M^G/C^) or homozygote (M^G/C^/M^G/C^) for the Gly1516Cys substitution located toward the C-terminus of the major collagenous domain in type XXVII collagen were viable, fertile and exhibited growth rates indistinguishable from their wild type (WT/WT) littermates (Fig. 2A). The only skeletal abnormality detected at birth was a fusion of the 4^th^ and 5^th^ sternebrae seen in M^G/C^/M^G/C^ mice (Fig. 2B). Skeletons from 9 week old mice of all genotypes appeared normal by X-ray analysis (Fig. 2C). Histological analyses of growth plates revealed that some M^G/C^/M^G/C^ mice displayed a slight disordering of the proliferative zone with limited clustering of cells (e.g. cf Fig. 3 B&E) whereas the growth plates of other M^G/C^/M^G/C^ mice appeared normal. Electron microscopic examination of the proliferative and hypertrophic chondrocytes and their surrounding ECM revealed no overt differences between control and mutant growth plates (Fig. S1). WT/M^G/C^ mice had no discernable growth plate phenotype (data not shown). The Gly to Cys mutation did not affect the secretion of collagen XXVII in the growth plate as judged by immuno-staining (Fig. 3 G–L).

### An 87 amino acid deletion in the type XXVII collagenous domain causes a perinatal lethal phenotype

**Figure 4:**
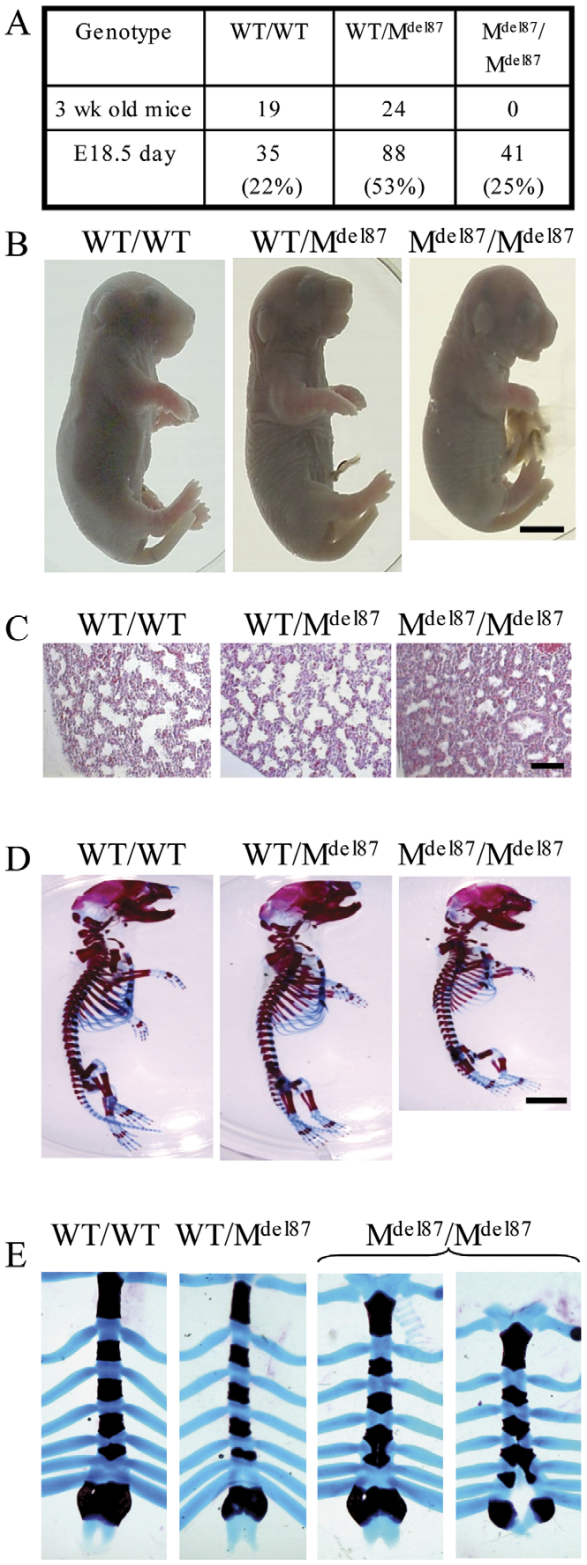
Gross phenotypic analyses of the Col27del87 mouse. A. Mice heterozygous for the Col27del87 allele were mated and offspring genotyped at 3 weeks of age revealing that litters contained wild type (WT/WT) and heterozygous (WT/M^Δ87^) mice but none homozygous (M^Δ87^/M^Δ87^) for the Col27del87 allele. However, at E18.5, M ^Δ87^/M^Δ87^ embryos were present in the normal Mendelian range. B. E18.5 M^Δ87^/M^Δ87^ embryos were smaller, had a domed head and shortened snout and developed a hernia at the umbilicus as a result of breaking the cord. WT/WT and WT/M^Δ87^ littermates were of normal size and appearance. Scale bar  =  3 mm. C. H+E stained sections of the lungs from E18.5 embryos carrying the Col27del87 allele revealed that M^ Δ87^/M^Δ87^ mice have hypercellular lungs when compared to WT/WT and WT/M^ Δ87^ littermates. Scale bar  =  100 µm. D. Gross analysis of the skeleton from Col27del87 mice reveals that M^ Δ87^/M^ Δ87^ mice were smaller than their WT/WT and WT/M^ Δ87^ littermates. Scale bar  =  3 mm. E. M^ Δ87^/M^ Δ87^ mice had either a fused 4^th^ and 5^th^ sternebrae or a cleft through the xiphoid complex up to the 4^th^ sternebrae.

The Col27del87 line was established by breeding the Col27G1516C mouse with a deletor cre line leading to the excision of exons 50 to 55 of the *Col27a1* gene (Fig. 1 E) and a resulting in-frame 87 amino acid deletion close to the C-terminus of the major collagenous domain. Mice heterozygous for this deletion (WT/M^Δ87^) were viable, fertile and had no overt phenotype. However no offspring homozygous for the mutation (M^Δ87^/M^Δ87^) were represented in litters at 3 weeks of age (Fig. 4 A). Litters of E18.5 embryos had the expected Mendelian ratio of genotypes including 25% M^Δ87^/M^Δ87^ pups (Fig. 4 A). E18.5 WT/WT and WT/M^Δ87^ pups that were delivered by Caesarian and warmed under a lamp breathed spontaneously whereas M^Δ87^/M^Δ87^ pups performed gasping spasms but failed to take an effective breath and were humanely sacrificed. We concluded that M^Δ87^/M^Δ87^animals died perinatally due to a breathing defect.

### M^Δ87^/M^Δ87^ embryos have a severe chondrodysplasia

E18.5 M^Δ87^/M^Δ87^ embryo's appeared marginally smaller than their littermates (mean±SEM [n]; WT/WT 1.34±0.09 g [6], WT/M^Δ87^ 1.41±0.04 g [21], M^Δ87^/M^Δ87^ 1.25±0.03 g [11]), had a slightly domed skull, a short snout, a receding lower jaw, shorter limbs and developed a midline hernia when the umbilical cord was removed (Fig. 4 B). WT/M^Δ87^ embryos were indistinguishable from their WT/WT littermates (Fig. 4 B). Histological examination revealed no defects in the diaphragm of E18.5 M^Δ87^/M^Δ87^ embryos (data not shown) although their lungs were hypercellular with poorly developed airspaces in comparison with WT/WT and WT/M^Δ87^ littermates (Fig. 4 C). The role of collagen XXVII in lung development is being examined as part of a related but separate study and will not be described further here. Skeletal preparations of E18.5 M^Δ87^/M^Δ87^ embryos exhibited shorter endochondral bones throughout the body whereas the WT/M^Δ87^ embryos appeared indistinguishable from WT/WT controls (Fig. 4 D). In addition, the M^Δ87^/M^Δ87^ embryos had sternal defects with either a fusion of sternebrae 4 and 5 and/or a clefting of the zyphoid resulting from a developmental failure of the two parts of the sternum to align and fuse correctly (Fig. 4 E).

**Figure 5:**
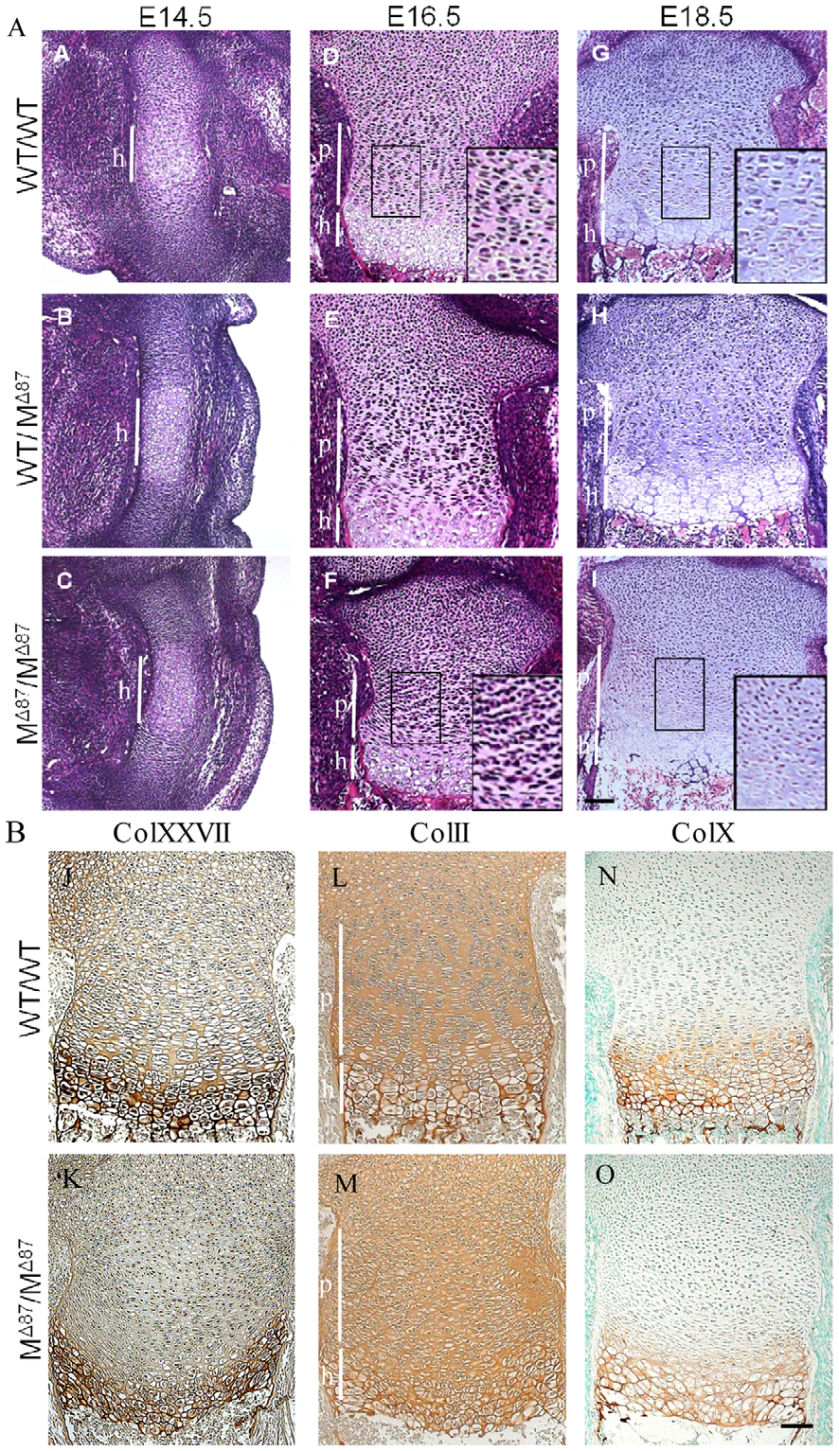
Tibial growth plate analyses from Col27del87 mice. A. The growth plate of the developing tibia of M^ Δ87^/M^ Δ87^ E14.5 embryos (C) appeared normal when compared to the WT/WT(A) and WT/M^ Δ87^ littermates (B). The proliferative zone (see insets) of M^ Δ87^/M^ Δ87^ E16.5 and E18.5 embryos (F and I, respectively) appeared highly disorganised when compared to the WT/WT (D and G, respectively) and WT/M^ Δ87^ littermates (E and H, respectively). Scale bar  = 100 µm. B. Immunolocalisation of collagen XXVII, collagen II and collagen X in E18.5 tibial growth plates. The staining for collagen XXVII in the proliferative zone of M^ Δ87^/M^ Δ87^ embryos (K) was reduced and the chondrocytes lacked a translucent pericellular zone compared to the WT/WT controls (J). The distribution of collagens II and X were similar in the control and mutant growth plates (L–O). Scale bar  =  100 µm.

Histological examination of developing bones revealed no apparent differences between the genotypes at E14.5 (Fig. 5 A–C). At E16.5, proliferative zone chondrocytes in WT/WT and WT/M^Δ87^ growth plates had adopted a discoid shape with a prominent translucent pericellular matrix (apparent after H&E staining) and formed longitudinal columns (Fig. 5 D and E). Chondrocytes in the equivalent region of the M^Δ87^/M^Δ87^ growth plate remained less flattened and a displayed reduced tendency to organise into columns or to develop a translucent pericellular region (Fig. 5F). These differences persisted at E18.5 (Fig. 5G–I). Despite the disruption to the proliferative zone, chondrocytes in the M^Δ87^/M^Δ87^ growth plates still underwent hypertrophy (Fig. 5I & O). Collagen XXVII secretion in the proliferative zone was reduced in the E18.5 M^Δ87^/M^Δ87^ growth plate in comparison with the wildtype control (Fig. 5J and K) and absence of a translucent pericellular matrix was particularly apparent with this immunostain (Fig. 5K). Collagen II and X deposition appeared comparable in WT/WT and M^Δ87^/M^Δ87^ growth plates (Fig. 5L–O). Despite the severe disruption to growth plate architecture, the expression of key differentiation markers in the growth plate such as FGFR3, PTH receptor, Indian Hedgehog and Patched 1 occurred normally in the M^Δ87^/M^Δ87^ growth plates (Fig. S2).

**Figure 6:**
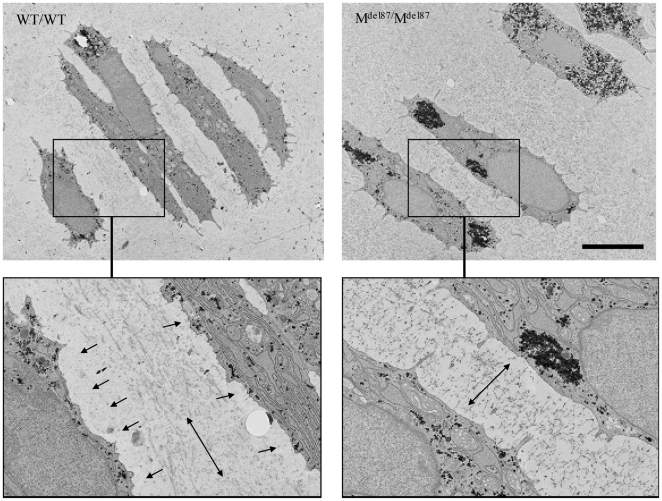
Ultrastructural analyses of the growth plate from the tibia of E18.5 day Col27del87 mouse. Transmission electron micrograph of proliferative zone chondrocytes from wild type (WT/WT) embryos and embryos homozygous for the mutation (M^ Δ87^/M^ Δ87^). The black granular material particularly evident in the cytoplasm of the M^Δ87^/M^Δ87^ sample is glycogen. Boxed areas are expanded below. Single-headed arrows indicate pericellular areas with relatively sparse staining present in the WT/WT but not mutant. Double headed arrows indicate the apparent alignment planes for the microfibrillar material deposited between the proliferative chondrocytes. Scale bar  =  5.0 µm.

Electron microscopic examination of the proliferative zone of E18.5 embryos revealed the expected stacks of discoid cells in the WT/WT E18.5 embryos (Fig. 6). The immediate pericellular space surrounding the chondrocytes was electron-translucent and devoid of the fine microfibrillar material that characterised the more distal ECM. In addition, the microfibrillar material between proliferative cells was aligned parallel to the plasma membranes in the WT/WT tissue (Fig. 6). In comparison, the proliferative zone cells in the E18.5 M^Δ87^/M^Δ87^ growth plates were less flattened. The microfibrillar material filled the space between adjacent cells and the microfibils were oriented perpendicular to the plasma membranes (Fig. 6). There was no obvious expansion or engorgement of the endoplasmic reticulum in the M^Δ87^/M^Δ87^ chondrocytes suggesting that protein misfolding and retention is not a significant factor in the resulting pathology.

### Mice with a cartilage-specific deletion in *Col27a1* are viable but have a severe chondrodysplasia

**Figure 7:**
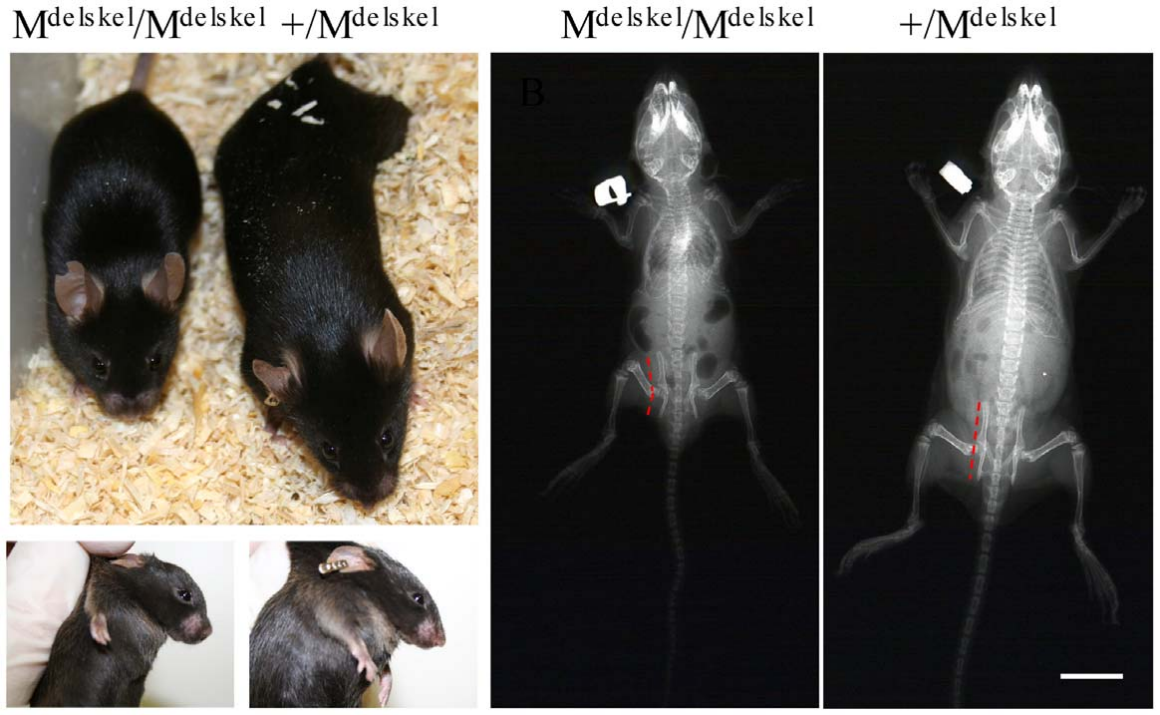
Appearance of mice carrying a cartilage-specific deletion in type XXVII collagen. Mice homozygous for a cartilage specific deletion of the collagen XXVII gene (M^Δskel^/M^Δskel^) were viable but were severely dwarfed. Left of figure – littermates at 4 weeks of age (+/M^Δskel^ is the phenotypically normal control). Right of figure - X-rays of 3 week old mice. M^Δskel^/M^Δskel^ mice exhibited a slight distortion of the thoracic cage, a mild thoracic kyphosis and a distortion of the pelvis (indicated by red dashed lines) compared to control littermates. Scale bar  =  10 mm.

The Col27delskel line was established by breeding the Col27G1516C mouse with a ColII cre line leading to the excision of exons 50 to 55 of the *Col27a1* gene (Fig. 1 G) within chondrocytes. Mice heterozygous for this cartilage-specific deletion on the Col27G1516C background (for simplicity referred to as +/M^Δskel^) were viable, fertile and had no discernable phenotype (Fig. 7). Mice homozygous for the cartilage specific deletion (M^Δskel^ /M^Δskel^) were viable and could breath normally demonstrating that the lung phenotype associated with the Col27del87 (M^Δ87^/M^Δ87^) genotype was not a secondary consequence of the cartilage defect. However, M^Δskel^/M^Δskel^ mice were strikingly dwarfed (Fig. 7) with affected mice often being only half the body weight of littermates and exhibiting a short snout and slightly domed skull (Fig. 7). X-rays revealed no overt skeletal patterning defects in the M^Δskel^/M^Δskel^ mice but all of the long bones were shorter than those in the littermate control (Fig. 7). M^Δskel^/M^Δskel^ mice also exhibited slight distortion of the thoracic cage, a mild thoracic kyphosis and a distortion of the pelvis (Fig. 7). M^Δskel^/M^Δskel^ males were fertile but the fertility of females was not tested due to their small body size.

### Growth plate chondrocytes and their pericellular matrix are disrupted in M^Δskel^/M^Δskel^ mice

**Figure 8:**
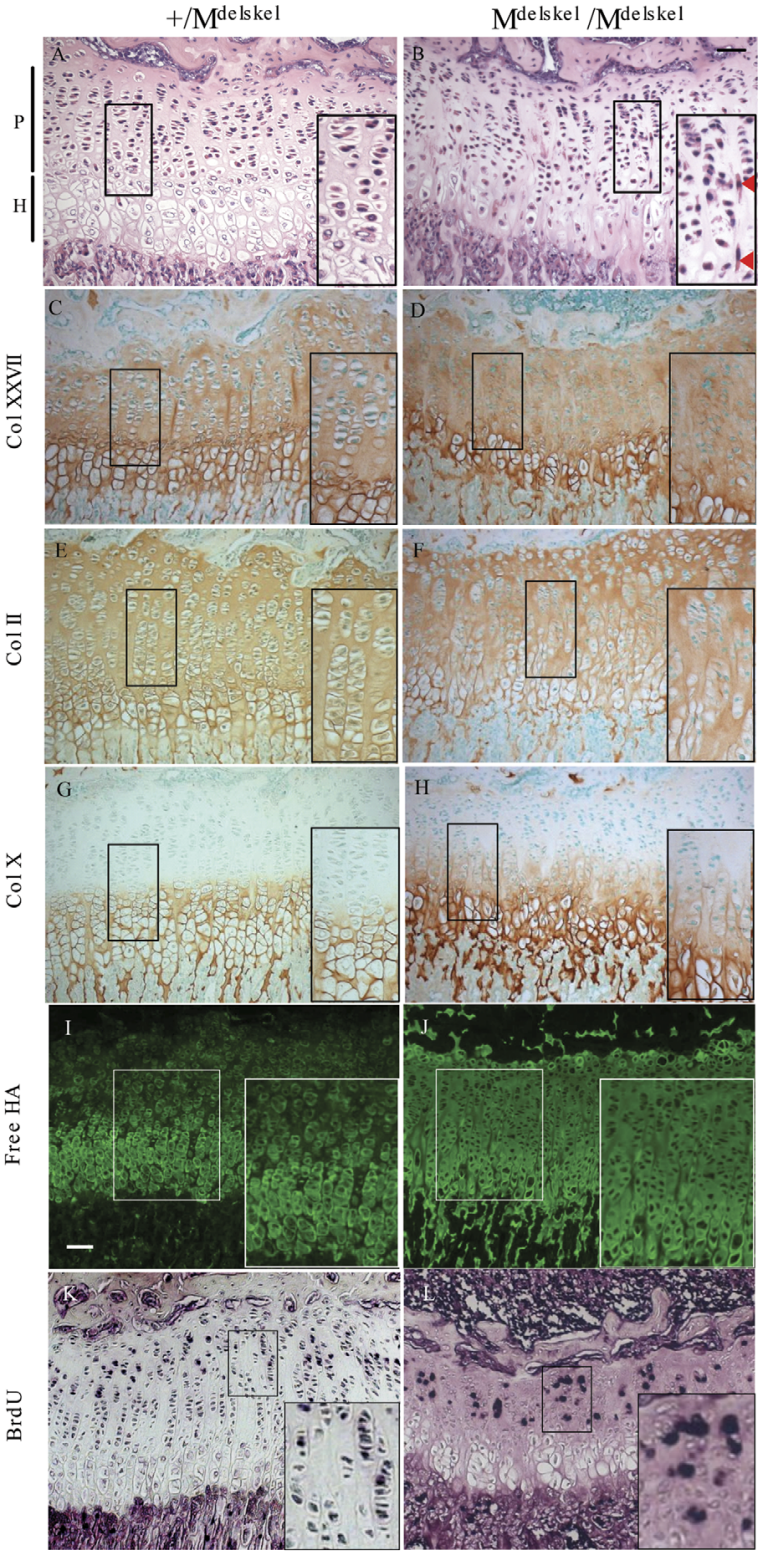
Histological analyses of 3 week old Col27delskel tibial growth plates. The organisation of proliferative zone chondrocytes in M^Δskel^ /M^Δskel^ mice (B) were highly disorganised and cells lacked the translucent pericellular matrix apparent in the phenotypically normal (+/M^Δskel^) littermates (A). Collagen XXVII immunostaining in control (C) and M^Δskel^ /M^Δskel^ (D) growth plates were of similar intensity but M^Δskel^ /M^Δskel^ lacked the stain-free pericellular zone apparent in controls. Expression of type II collagen in mice homozygous for the Col27delskel allele (F) appeared normal when compared to the control littermates (E). Collagen X immunostain was limited to the hypertrophic zone in controls (G) but appeared to extend into lower part of the proliferative zone in M^Δskel^ /M^Δskel^ mutant growth plates (H). Free hyaluronan was localized to the pericellular matrix in controls (I) but was more diffusely distributed in M^Δskel^ /M^Δskel^ growth plates (J). BrdU labeling of proliferative zone chondrocytes in control (K) and M^Δskel^ /M^Δskel^ growth plates (L). The disorganization and failure of the cells to from efficiently form columns is apparent in the mutant growth plate (L).

The growth plates of perinatal M^Δskel^/M^Δskel^ mice were severely disrupted compared to littermate controls but the proliferative chondrocytes displayed some signs of flattening and organising into vertical columns (Fig. S3). At 3 weeks, the secondary centre of ossification in tibias and femurs of M^Δskel^/M^Δskel^ mice was indistinguishable from that of controls (data not shown). The proliferative zone chondrocytes in the 3 week old M^Δskel^/M^Δskel^ mouse growth plates were adopting a flattened appearance and attempting to organise into vertical columns although the architecture remained disrupted in comparison with controls (Fig. 8A–B). In some instances, flattened proliferative chondrocytes were orientated at right angles to their normal plane and located between the vertical stacks of cells in the M^Δskel^/M^Δskel^ growth plates (Fig. 8B). In these 3 week old mice, the level of immunostaining for collagen XXVII appeared equivalent in the control and M^Δskel^/M^Δskel^ growth plates. However, collagen XXVII staining extended right up to the cells in the proliferative zone of M^Δskel^/M^Δskel^ growth plates and these lacked the translucent stain-free pericellular matrix of the littermate controls (Fig. 8 C–D). Collagen II immunostaining was similar in the control and M^Δskel^/M^Δskel^ growth plates although there was some evidence for localisation of limited staining within the proliferative chondrocyte pericellular matrix of the mutant which was not apparent in controls (Fig. 8 E–F). Collagen X localisation appeared relatively normal in hypertrophic zone of M^Δskel^/M^Δskel^ growth plates although there was evidence for some collagen X immunostain in the lower part of the proliferative zone in the mutant animals (Fig. 8 G–H). In control sections, free hyaluronan was localised predominantly to the pericellular matrix of hypertrophic chondrocytes. However, in M^Δskel^/M^Δskel^ growth plates, the free hyaluronan was more widely distributed in both the hypertrophic and proliferative zones (Fig. 8 I–J). BrdU analysis of proliferation rates revealed no significant differences in proliferation rates between the control and M^Δskel^/M^Δskel^ growth plates (mean±SEM[n] +/ M^Δskel^ 11.5±1.5% [3] vs M^Δskel^/M^Δskel^ 13.0±3.2% [3]) although the disorganization of the proliferating chondrocytes in the M^Δskel^/M^Δskel^ growth plate was apparent in comparison with controls (Fig. 8 K–L).

## Discussion

The gene targeting strategy adopted for this study enabled us to generate two distinct mutations in the collagen XXVII gene from a single targeting vector with the conversion of the missense Gly to Cys mutation to an 87 residue deletion within the collagenous domain triggered *in vivo* by the expression of cre recombinase.

The phenotypic consequences of the Gly to Cys mutation located 87 residues from the C terminus of the collagenous domain were unexpectedly mild. Such mutations toward the C-terminus of the classical fibrillar collagen genes are usually associated with moderate to severe disease phenotypes caused by a combination of decreased secretion resulting from delayed and misfolding of the collagenous domain accompanied by protein degradation in addition to defects in fibrillogenesis if the mutant protein is secreted into the extracellular compartment [11], [12]. However, collagen XXVII seems to be able to function relatively normally with this missense mutation in that whether heterozygous or homozygous for the mutation, animals grew normally with little disruption of growth plate architecture or collagen XXVII secretion. The only obvious skeletal abnormality in the M^G/C^/M^G/C^ animals was the fusion of two sternebrae, a relatively common type of defect that was also apparent in the M^Δ87^/M^Δ87^ mice (see below). The relatively milder than anticipated effects of the Gly to Cys mutation in collagen XXVII are likely due to two factors. Firstly, collagen XXVII has two conserved interruptions in the triple helical domain which indicates that the function of this fibrillar collagen is not dependent upon a perfect Gly –X –Y repeat in the collagenous domain [4], [5]. Hence collagen XXVII may have some capacity to accommodate small interruptions without significant effects upon folding and secretion. Secondly, collagen XXVII appears to assemble into very thin fibrils in the ECM that are independent of the thicker fibrils formed by the classical fibrillar collagens [7], [8]. Again these thin fibrils may be better able to accommodate mutant collagen trimers.

In contrast, the phenotypic effects of an 87 amino acid deletion within the C-terminal portion of the type XXVII collagen triple helical domain were severe in mice homozygous for the mutation (M^Δ87^/M^Δ87^ ) which died at birth due to a lung defect accompanied by chondrodysplasia and midline defects including a similar sterna defect to that described above in the M^G/C^/M^G/C^ mice. Collagen XXVII clearly plays a subtle role in the development and fusion events forming the sternum and involving the midline but extensive histological examination failed to reveal any overt reason for the abdominal weakness and the diaphragm was intact in the M^Δ87^/M^Δ87^ embryos.

The growth plate proliferative zone defect caused by the 87 amino acid deletion in the collagenous domain of collagen XXVII was apparent from E16.5 in the M^Δ87^/M^Δ87^ embryos and was characterized postnatally in the M^Δskel^/M^Δskel^ mouse. The first apparent changes in the E16.5 day proliferative zone involved a reduced tendency of these chondrocytes to flatten and organise into vertical columns accompanied by a disruption to the pericellular matrix (Fig. 5) that was also apparent by EM (Fig. 6). Postnatally, the characteristic features of proliferative zone chondrocytes (column formation and cell flattening) became more apparent in the M^Δskel^/M^Δskel^ mice but accompanied by obvious signs of a proliferative zone defect including misaligned cells and a disruption of the pericellular matrix. Furthermore, the diffusion of collagen X into the lower portion of the proliferative zone of the mutant growth plate again suggests a disruption of the ECM in the proliferative zone (Fig. 8). It is of note that the expression of the del 87 form of collagen XXVII did not alter the expression of key differentiation markers within the disorganized growth plate (Fig. S2) and proliferation rates were unaffected. The collagen XXVII mutation therefore appears to impact directly upon the organisation of the ECM of the proliferative zone rather than by affecting the signaling events controlling the differentiation process. The decrease in bone growth rate in the M^Δskel^/M^Δskel^ mouse is therefore most likely a direct consequence of pericellular matrix disruption leading to disordered proliferation and failure to form columns efficiently. Similar changes in proliferative chondrocytes including a disruption in the structure of the pericellular matrix, occasional mis-oriented flattened chondrocytes between the columns of proliferative cells in older animals (Fig. 8B) and diffusion of collagen X into the lower part of the proliferative zone have been noted in the collagen IX knockout mouse [13 and Attila Aszodi personal communication]. These pathological features have been attributed to altered interactions between the proliferative chondrocyte and the pericellular matrix that are required for proliferative chondrocyte flattening and correct alignment of these cells into columns [14]. The collagen XXVII sequence does not contain proven integrin binding sites but does contain a GLOGEO and a GLOGDA sequence (where O is predicted to be hydroxyproline) which represent potential binding motifs based on homologies to known sites in the major fibrillar collagens (R. Farndale, personal communication). It is conceivable that the 87 amino acid deletion, which is C-terminal to the putative integrin-binging motifs and the resulting disruption in the triple helical structure of collagen XXVII may interfere with the chondrocyte's ability to bind collagen XXVII. Alternatively, the disorder in the proliferative zone ECM may be caused by altered interactions of the mutant collagen XXVII with other matrix components.

The knockdown of collagen XXVII isomers in zebrafish embryos produced the formation of notochord curves at the distal end of the tail and misshapen vertebrae [15] demonstrating a clear role for this collagen in skeletogenesis. The mutant mice expressing the del87 allele generated in this study did not show obviously misshapen vertebrae but exhibited a severe chondrodysplasia including a mild kyphosis, and a clear growth plate defect. These overlapping but distinct effects on skeletogenesis may reflect species differences or alternatively originate in the different strategies utilized (knockdown in the fish versus the expression of mutant forms of the protein in the current study).

Through the application of a conditional knock-in strategy, we have been able to determine the effects of two distinct mutations in collagen XXVII on endochondral bone growth. The Gly to Cys mutation within the C-terminal region of the collagenous domain produced a very mild phenotype whereas the much larger 87 amino acid residue in-frame deletion in the collagenous domain not only disrupted endochondral ossification but also caused defects in other tissues including lung. Interesting, neither collagen XXVII mutation caused any overt phenotype in articular cartilage. It was anticipated that the del87 allele would produce a hypomorph or functional null for collagen XXVII due to misfolding of the triple helical domain and subsequent intracellular protein degradation by way of ER associated degradation or autophagy [16]. In some ways, the most surprising finding was that the mice heterozygote for either the Gly to Cys or the del87 mutation had no phenotype and appeared to secrete collagen XXVII efficiently based on immunolocalisation. In a heterozygote, the trimerisation of mutant and wild type proα1(XXVII) chains at the carboxyl- terminal non-collagenous domain should result in 87.5% of trimers containing at least one mutant chain. In the classical fibrillar collagens, this is sufficient to cause significant consequences either in terms of protein misfolding and reduced secretion or if secreted, in the disruption of the resulting fibril structure. Type XXVII collagen forms very thin fibrils that are distinct from the thicker fibrils formed by types II and XI collagen in cartilage [7], [8]. The lack of phenotype in terms of growth plate function and immunostaining in heterozygotes for the collagen XXVII missense or deletion mutation suggests that this collagen is far more tolerant of sequence change compared to its classical counterparts. Furthermore, the phenotypic effects of the Gly to Cys mutation were extremely mild with significant pathology only becoming apparent with a much larger deletion in the collagenous domain.

Unfortunately, we have been unable to detect quantifiable levels of collagen XXVII in tissue extracts using western blotting and so have been unable to confirm the levels of secretion and molecular form of the molecule secreted in control and mutant cartilage by biochemical means. Nevertheless, these results suggest disease associated mutations in collagen XXVII may be less common than one would predict based on extensive studies of classical fibrillar collagens [e.g. 11,12]. Human disease resulting from mutations in collagen XXVII are likely to result more commonly from larger deletions in the collagen XXVII gene rather than missense mutations and may prove perinatal lethal involving not only chondrodysplasia but also problems in other tissues such as lung and midline defects.

In summary, collagen XXVII plays an important role in organizing the pericellular ECM of proliferative zone chondrocytes. Without the correct organisation of this matrix, the pattern of cell division and the ability of the proliferative chondrocyte to correctly organise into columns are disrupted resulting in a significant decrease in the rate of endochondral bone growth.

## Materials and Methods

### Ethics statement

The mouse work described here was approved by the University Animal Ethical Review Group and conducted under a project licence (Animals [Scientific Procedures] Act 1986) granted by the UK Home Office.

### Generation of Collagen XXVII mutant mice

Gene targeting was performed as described [17]. Briefly, a PAC clone was identified and ordered from HGMP (Hinxton, UK: at http://www.hgmp.mrc.ac.uk) using a 129/sv DNA library (RPCI-21) supplied as filters and screened with a cDNA encoding the C-terminus of collagen. The 10 kb *Eco RV* fragment containing the 3′ region of the collagen XXVII was sub-cloned into pBluescript (pBS). The 1 kb *Sac 1- Kpn 1* fragment encoding exon 50 and flanking introns was used to produce the G/T transition introducing a glycine to cysteine substitution near the C-terminus of the collagenous domain of collagen XXVII (Gly1516Cys). The QuikChange XL site directed mutagenesis kit (Stratagene) was used together with the following forward and reverse mutagenic primers: CCCCACAGGGTGACTCTTGCGAGATGGGCfTTCCCAGGAGTGGC and GCCACTCCTGGGAAGCCCATCTCGCAAGAGTCACCCTGTGGGG respectively. The presence of the mutation was confirmed by sequencing. A lone *lox*P site was inserted into the Sac1 site 5′ to exon 50 and the floxed *Neo TK* selection cassette inserted at the *Xho 1* site 3′ of exon 55 (Fig.1). The targeting construct was linearized with *Not* I and electroporated into R1 (129Sv) ES cells (http://www.mshri.on.ca/nagy/index.html). Cells were then grown in medium containing G418 (500 µg/ml) for 5–6 days and 360 resistant clones picked and cultured for freezing down and DNA isolation. ES clone DNA was screened for homologous recombination by *Eco* RV digestion and Southern blot analysis using the external probe indicated (Fig.1). The presence of the mutation in homologously recombined clones was confirmed by PCR detection of the *lox* P site 5′ of exon 50 and by direct sequencing of PCR products spanning exon 50 of the *Col27a1* gene. Homologously recombined clones were then grown and transiently transfected with the *cre* recombinase gene by electroporation as described previously [18] to remove the floxed *Neo* TK selection cassette. Correctly targeted clones were used to generate germ-line chimera's [17] from which the Gly1516Cys line was established. The Col27del87 and Col27delskel (del87 deletion present only in chondrocytes) lines were subsequently produced by crossing the Gly1516Cys line with a deletor *cre* [19] and colII *cre* line [20] respectively. Two separately targeted ES cell clones containing the Col27G1516C allele were used to generate germline chimeras. Both lines gave similar phenotypes for the Col27G1516C and Col27del87 alleles. Unless otherwise stated, at least 3 mice of each genotype were analysed.

Genotyping for WT (primers a & b), col27G1516Cys (primers a & b) and Col27del87 (primers a and c) alleles was performed by PCR on tail DNA using the following primers (see Fig. 1) a: GTCAGGAAACTGTGCTTTATAG; b: GGAAAGCAAGGCTTGTATAC; c: ACATGGATGGGACTCTTGCT.

### Growth curves and skeletal analysis

Growth curves were produced by weighing at specified times and the data analysed by one-way ANOVA. X rays of adult mice were produced using a Flaxitron X ray specimen radiography system. Skeletal preparations of E18.5 embryo or newborn mice were prepared as described previously [21].

### Histology and immunohistochemistry

Tissue samples from mice of defined genotypes were fixed overnight in either 95% ethanol/5% acetic acid or ice-cold 10% formalin. Bone samples were decalcified in 0.25 M EDTA before embedding in paraffin wax and sectioning. Immunohistochemistry was carried out on ethanol/acetic acid-fixed samples using antibodies against collagen XXVII [7], collagen X [22], collagen II (Abcam), and BrdU (Abcam) as described previously [7]. BrdU labeling of proliferating cell nuclei was carried out as described [18]. To visualise free hyaluronan, sections were probed with 5 µg/ml biotinylated hyaluronan-binding protein (Cosmo Bio Co Ltd., Tokyo, Japan) in blocking solution (2% (v/v) foetal calf serum/PBS) overnight at 4°C, followed by washing in PBS and incubation in 40 µg/ml streptavidin conjugated to Alexa Fluor-488 (Invitrogen, Paisley, UK) in blocking solution for 30 min at room temperature. Sections were then washed in PBS three times before mounting with Vectashield® mounting medium containing DAPI counterstain (Vector Laboratories Ltd., Peterborough, UK) to visualise nuclei.

### In situ analysis

In situ hybridisation using ^35^S-labelled RNA probes was carried out as described [23] on E18.5 embryos and 3 week old tibias (from animals with the genotypes specified) that had been fixed in ice-cold buffered paraformaldehyde, embedded in paraffin wax and sectioned in a saggital plane. In situ probes for collagen XXVII [5] and collagen X [22] were as described previously. For the following genes, cDNA inserts from the indicated IMAGE clones were subcloned into the pT7T3 vector and antisense probes generated from appropriately linearised vectors: PPR (PTHrP receptor) CloneID 3169139, FGFR3 CloneID 5708838, IHH CloneID 6395758, PTCH1 Clone ID6509228.

### Light microscopy

All light microscopic images were collected using a Zeiss Axiovision microscope fitted with an Axiocam CCD camera.

### Ultrastructural analysis

Tibia were fixed overnight in 4% formaldehyde/2.5% gluteraldehyde in 0.1 M sodium cacodylate buffer, followed by three washes in 0.1 M sodium cacodylate buffer. Samples were then incubated in a secondary fix of 1% osmium tetroxide for 2 h at 48°C, followed by three washes in water. Staining of the sample was carried out by incubation in 0.5% uranyl acetate for 1 h at 48°C, followed by water washes. The samples were then dehydrated through an ascending graded acetone series. The acetone was replaced with two changes of propylene oxide, which in turn was replaced with Spurr's resin. After several changes, the resin was polymerized by incubating at 60°C for 48 h. Sections of 70 nm were cut and stained with 0.3% (w/v) lead citrate. Images were taken on a FEI Tecnai 12 Biotwin electron microscope, recorded on 4489 film (Kodak) and scanned using an Imacon Flextight 848 scanner (Precision Camera and Video).

Acknowledgements: The authors thank the staff in the EM facility in the Faculty of Life Sciences for their assistance, and the Wellcome Trust for equipment grant support to the EM facility.

## Supporting Information

Figure S1 **Transmission EM of growth plate from newborn Col27G1516C mouse.** 2 µm thick epoxy-embedded sections were stained with toluidine blue and examined by light microscopy (LM) for orientation purposes. 70 nm sections were stained as described in methods and examined by transmission EM.
(TIF)

Figure S2 **Isotopic** ***in situ*** **hybridisation of molecules involved in the major signalling pathways controlling growth plate differentiation.** In the tibia of E18.5 day embryos homozygous for the Col27del87 allele, collagen XXVII (M, S), PPR (N, T), collagen X (O, U), FGFR3 (P, V), IHH (Q, W), PTCH1 (R, X) appear unaffected when compared to the wild type littermates (A, G; B, H; C, I; D, J; E, K; F, L; respectively). G-R, dark field; A-F:S-X, bright field. Bar  =  100 µm. Wild type (WT/WT), Homozygous (M/M).
(TIF)

Figure S3 **Tibial growth plates from perinatal M^Δskel^ /M^Δskel^ mice.** H&E stained growth plates from phenotypically normal control (+ /MΔskel) and M^Δskel^ /M^Δskel^ mice. Growth plates from 3 week old equivalents (included in Fig. 8) are shown for comparison.
(TIF)
