# Supplementary figures and images for: Collagen XXVII Organises the Pericellular Matrix in the Growth Plate
Source: PLoS One. 2011 Dec 19;6(12):e29422. doi: 10.1371/journal.pone.0029422 (PMC3242791; doi:10.1371/journal.pone.0029422)

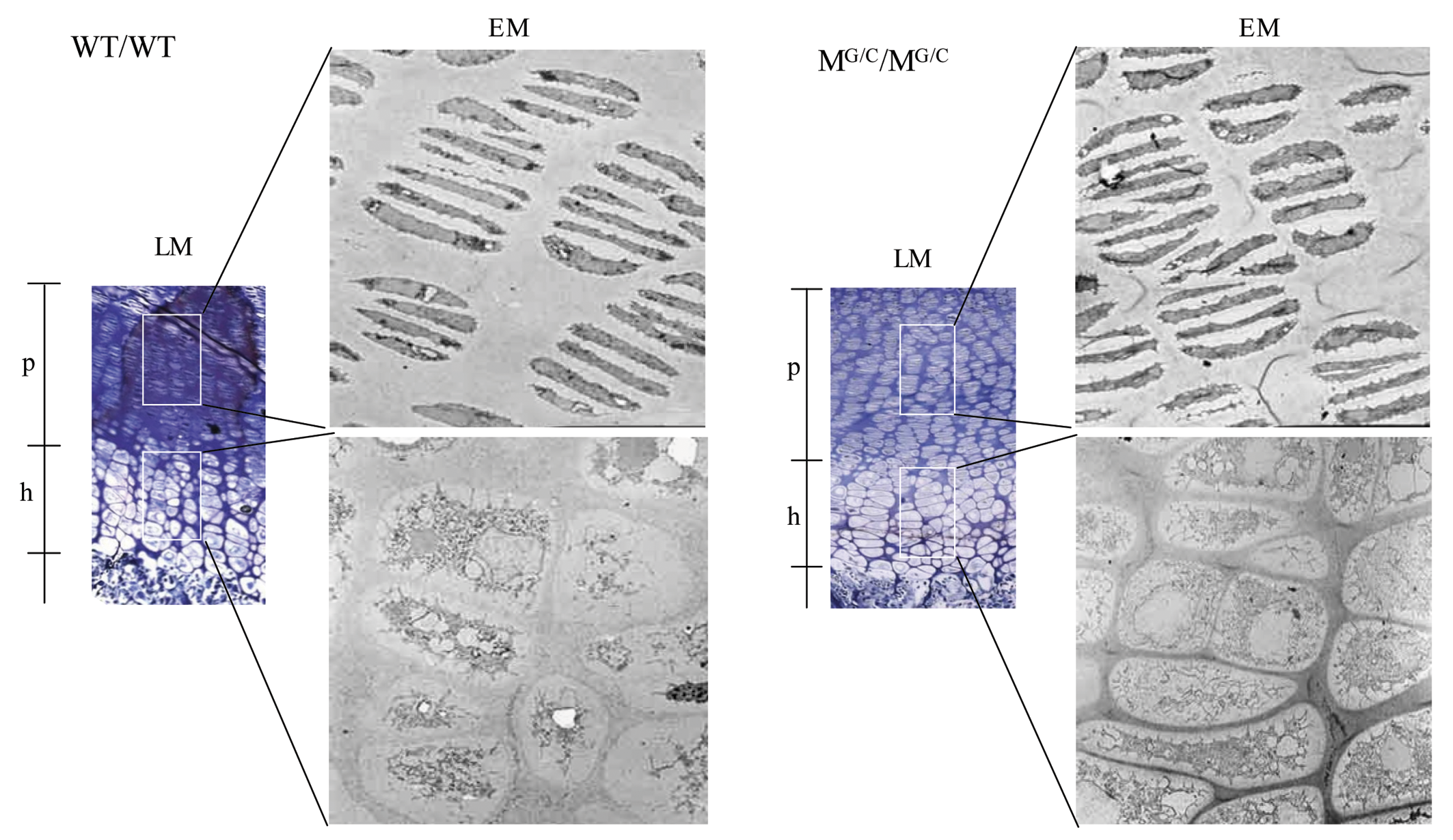

Supplement: Figure S1 — Transmission EM of growth plate from newborn Col27G1516C mouse. 2 µm thick epoxy-embedded sections were stained with toluidine blue and examined by light microscopy (LM) for orientation purposes. 70 nm sections were stained as described in methods and examined by transmission EM. (TIF) [file pone.0029422.s001.tif]

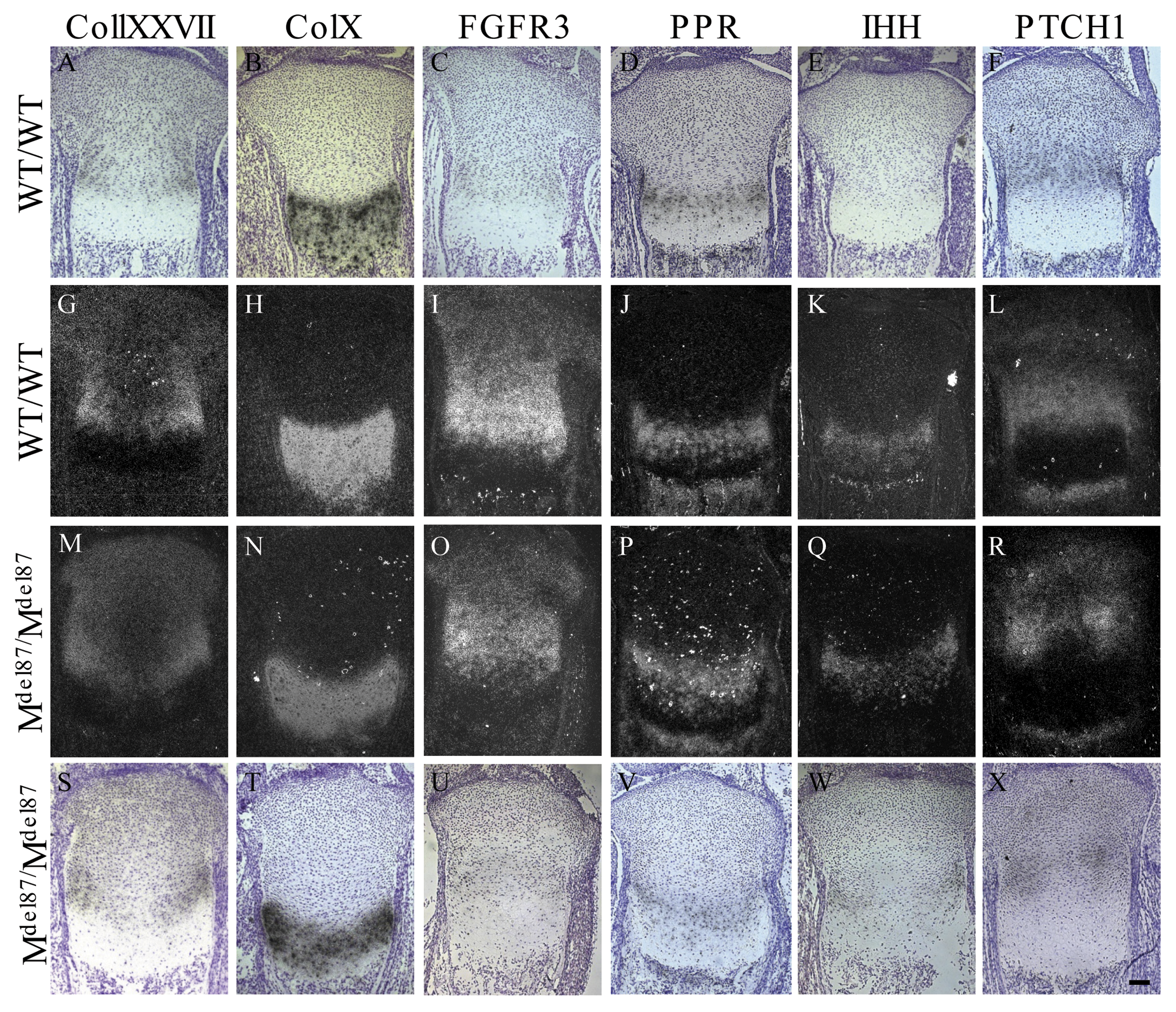

Supplement: Figure S2 — Isotopic in situ hybridisation of molecules involved in the major signalling pathways controlling growth plate differentiation. In the tibia of E18.5 day embryos homozygous for the Col27del87 allele, collagen XXVII (M, S), PPR (N, T), collagen X (O, U), FGFR3 (P, V), IHH (Q, W), PTCH1 (R, X) appear unaffected when compared to the wild type littermates (A, G; B, H; C, I; D, J; E, K; F, L; respectively). G-R, dark field; A-F:S-X, bright field. Bar = 100 µm. Wild type (WT/WT), Homozygous (M/M). (TIF) [file pone.0029422.s002.tif]

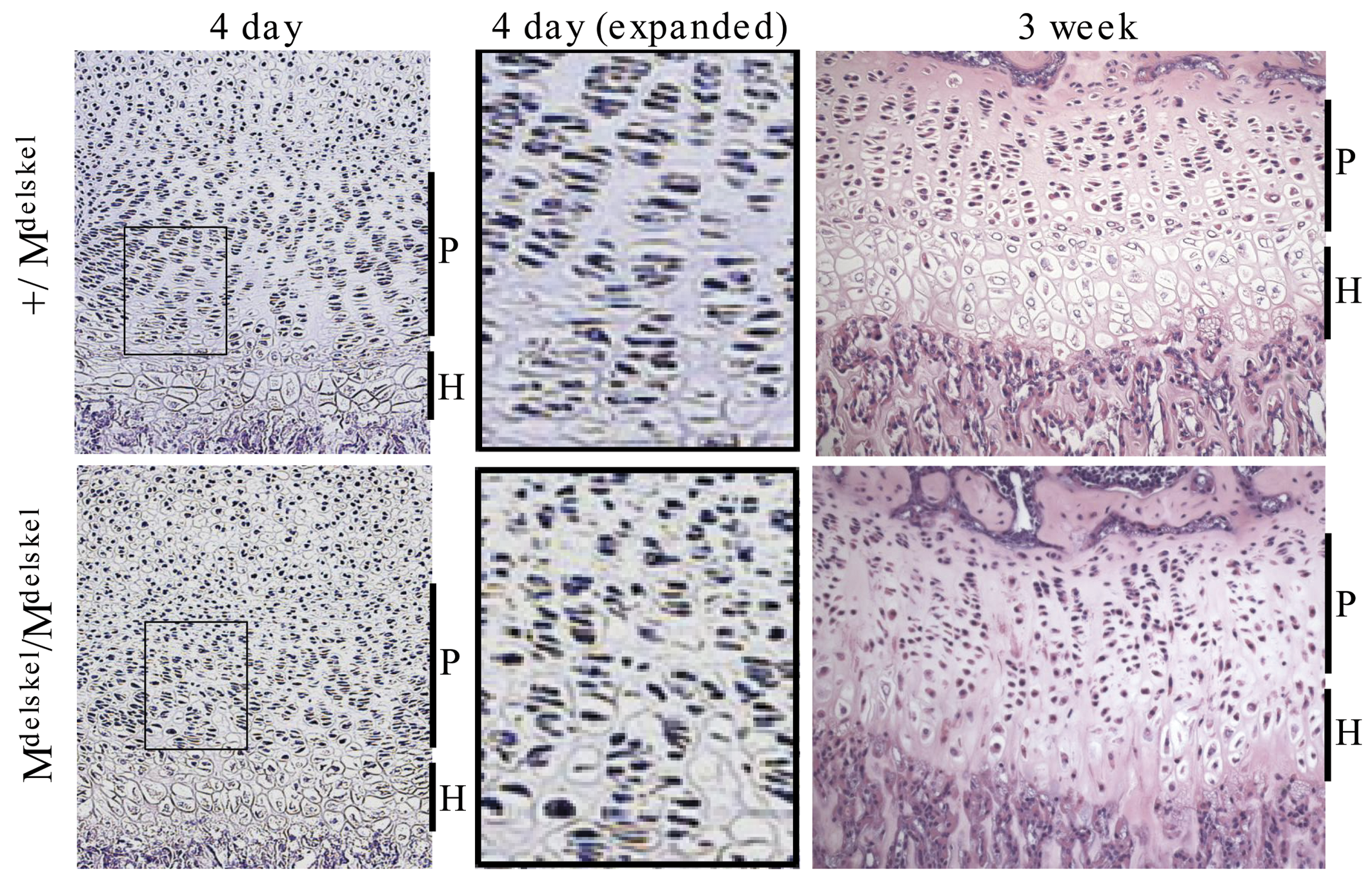

Supplement: Figure S3 — Tibial growth plates from perinatal MΔskel /MΔskel mice. H&E stained growth plates from phenotypically normal control (+ /MΔskel) and MΔskel /MΔskel mice. Growth plates from 3 week old equivalents (included in Fig. 8) are shown for comparison. (TIF) [file pone.0029422.s003.tif]
